# *Plasmodium falciparum* histidine rich protein-2 diversity and the implications for PfHRP 2: based malaria rapid diagnostic tests in Ghana

**DOI:** 10.1186/s12936-016-1159-z

**Published:** 2016-02-18

**Authors:** Linda Eva Amoah, Joana Abankwa, Akua Oppong

**Affiliations:** Noguchi Memorial Institute for Medical Research, University of Ghana, Legon, Ghana

**Keywords:** Malaria diagnosis, *Plasmodium falciparum*, Rapid diagnostic test (RDT), *Plasmodium falciparum* histidine rich protein-2 (PfHRP-2), *Plasmodium falciparum* histidine rich protein-3 gene (*pfhrp*3)

## Abstract

**Background:**

Malaria rapid diagnostic tests (RDTs) play a key role in malaria management and control. The PfHRP-2 based RDT is the most widely used RDT for malaria diagnosis in Ghana. Deletion of *pfhrp*2 in *Plasmodium falciparum* parasites affect the diagnostic accuracy of PfHRP-2 based RDT kits. Identifying the prevalence and distribution of *P. falciparum* parasites with deleted *pfhrp*2 is important for malaria control.

**Aim:**

The purpose of this study was to identify and confirm the prevalence of *pfhrp*2 deletant *P. falciparu*m parasites circulating within different regions of Ghana.

**Methods:**

DNA was extracted from the membrane of spent CareStart™ PfHRP-2 RDT kits and dried filter paper blood blots using Chelex-100. Exon 2 of *pfhrp*2 and *pfhrp*3 genes were amplified by polymerase chain reaction (PCR), resolved by agarose gel electrophoresis and visualized under UV light.

**Results:**

Microscopic analysis of blood smears from samples that were PfHRP-2 RDT positive revealed a parasite prevalence of 54/114 (47.4 %) and 2/26 (7.7 %) in Accra and Cape Coast, respectively. PCR analysis increased parasite prevalence in the RDT positive samples to 94/114 (82.5 %) and 6/26 (23.1 %) in Accra and Cape Coast respectively. The exon 2 of the *pfhrp*2 gene was deleted in 18/54 (33.3 %) of the microscopy confirmed and 36.2 % (34/94) of the PCR confirmed RDT positive samples collected in Accra. No RDT sample, confirmed to contain parasites by either PCR or microscopy was negative by *pfhrp*2 exon 2 PCR in Cape Coast. A survey of an additional 558 DBS revealed that 22.4 % (46/205) and 40 % (44/110) of PCR positive samples in Accra and Cape Coast, respectively, lacked the exon 2 region of *pfhrp*2 and possibly the entire *pfhrp*2 gene.

**Conclusions:**

A high number of *P. falciparum* parasites, which lack *pfhrp*2 exon 2 gene have been identified in two communities in Ghana. Continuous nationwide monitoring of the prevalence of *pfhrp*2 deletant parasites would be essential to malaria control. The use of RDT kits that are effective at malaria diagnosis despite deletion of *pfhrp*2, such as the PfHRP-2/PfLDH combo RDT kit could enhance the diagnosis of clinical malaria in Ghana.

## Background

Malaria is one of the deadliest infectious diseases of humanity, which causes significant mortality and morbidity in the tropics, particularly in Africa [1]. Malaria is a parasitic disease transmitted through the bite of an infectious female *Anopheles* mosquito. Early diagnosis is very important for disease management and the effective treatment of malaria. Before the advent of malaria rapid diagnostic tests (RDTs), diagnosis was based on microscopy of thick blood smears, which is still the gold standard for malaria diagnosis. However, in a number of rural and semi urban settings where lack of equipment, trained personnel and electricity prevents this essential diagnosis, health practitioners diagnose malaria based solely on clinical evaluation of symptoms [2]. RDTs offer a great potential for rapid immediate diagnosis of malaria infections, which has led to prompt and appropriate treatment of the disease, particularly in highly endemic rural settings [3].

Presently there is a very large demand for malaria RDT kits, as the World Health Organization (WHO) has recommended its use and majority of National Malaria Control Programmes have accepted it as the first step in the diagnosis of malaria. Due to the importance of the results of this initial screen, the WHO has established two programmes, the Foundation for Innovative New Diagnostics (FIND) malaria RDT quality assurance programme and WHO-FIND malaria RDT lot testing programme whose main mandate are to ensure accurate diagnosis of malaria [4]. Malaria RDT kits are designed to detect either *Plasmodium falciparum* specifically or discriminately detect both *P. falciparum* in addition to another human malaria parasite or indiscriminately detect all human malaria parasites [4, 5]. The main antigens that malaria RDT kits detect are PfHRP2, parasite lactate dehydrogenase (pLDH), and parasite aldolase (pAldo). PfHRP-2 is a *P. falciparum* specific antigen with the advantage of being highly abundant and heat stable however, the PfHRP-2 antigen remains in circulation for up to 4 weeks after the malaria parasites have cleared [6, 7]. Some monoclonal antibodies directed against PfHRP-2 have been found to cross react with PfHRP-3, a structural homologue of PfHRP-2 [8, 9]. Thus although PfHRP-2 based RDT kits have the highest sensitivities [4], they also have high false positive rates. By 2015, 171 different malaria RDT products had been tested by the WHO. Forty-five of these products detect only *P. falciparum*, ten detect *P. falciparum* as a part of a mixed infection with other human malaria parasites, one is *Plasmodium vivax* specific and 115 detect and distinguishing *P. falciparum* from either *P. vivax* mixed infections or mixed infections containing all the other human malaria parasites, *P. vivax*, *P. ovale* and *P.malariae* [10].

The accuracy of malaria RDT results can be affected by test antibody stability, product design and quality as well as the transport and storage conditions of the kits and sample parasite density [10]. Accurate diagnosis of malaria by PfHRP-2 RDT kits can be affected by the *pfhrp*2 and or *pfhrp*3 genotype of the parasite [5, 10, 11], the amount of PfHRP-2 antigen produced by the parasite [12, 13] as well as the longevity of PfHRP-2 antigen after parasite clearance. One major obstacle in the diagnosis of malaria by RDT, without additional confirmation of parasitaemia is false positive test results, which leads to the unnecessary administration of anti-malarial drugs when no malaria parasites are actually present in the patient. False positive RDT test results are frequently obtained immediately following an anti-malarial drug regimen, when parasites are cleared or densities very low, but the antigen remains in circulation weeks later [14, 15].

In some facilities in Ghana, where microscopy is unavailable, malaria is treated based on RDT results. It is thus very important to monitor the accuracy of RDT results as well as identify factors that affect the diagnostic ability of malaria RDTs. So far the main studies conducted in Ghana have determined the sensitivity and specificity of different brands of malaria RDT kits, including the CareStart™ and Paracheck RDT Kit [13, 16–18], in different cohorts of malaria This study systematically identifies and confirms the presence of *pfhrp*2 deletant (*pfhrp*2−) parasites as well determines the prevalence of *P. falciparum* parasites with deletions in *pfhrp*2 and *pfhrp*3 (*pfhrp*3−) in two communities in Ghana.

## Methods

### Ethics, consent and permissions

This study was approved by the Institutional Review Board (IRB) of the Noguchi Memorial Institute for Medical Research, University of Ghana. Prior to enrollment, the study was explained to all participants after which written informed consent was obtained. Parental consent was obtained from parents and guardians of all children in addition to child assent obtained from children between 12 and 17 years.

### Study area and sample collection

Abura Dunkwa, also known as Abura, is the district capital for Abura-Asebu-Kwamankese district and the Cape Coast Metropolis of the Central Region with a rural population of 31,768 for children under 14 years of age [19]. The Central Region is situated 165 km west of Accra (capital of Ghana). Malaria peak season coincides with the major rainy season between June through August. The community is a farming community.

Obom is in the Ga south municipality of the Greater Accra Region with a rural population of 22,368 for children under 14 years of age [20]. Malaria is perennial although it increases during the peak rainy season from June to August. The community is a fishing community. In 2014, malaria was estimated by microscopy to account for 35 % of all out patient visits at the local Obom health centre.

The study utilized a total of 226 spent PfHRP-2 based RDT kits as well as 558 filter paper blood blots from consenting healthy children within the two study sites in 2015. Thick and thin blood smears as well as DBS samples were obtained from healthy school children as part of a monthly malaria-screening programme from February through May. In April, RDT was performed according to manufacturers instructions in addition to the DBS and blood smears. The spent RDT cassettes were stored at room temperature for a maximum of 1 week, after which their membranes were processed for DNA. Approximately 50 μl of finger-pricked blood was spotted on to filter paper to make the DBS and thick blood smears. DBS were kept in sealed plastic bags with a desiccant and stored at −20 °C for no longer than 2 weeks after which they were processed for genomic DNA (gDNA). The slides containing the thick and thin blood smears were air dried and stored in slides boxes.

A sample was defined as negative by microscopy when two independent microscopists confirmed the absence of *P. falciparum* parasites on a Giemsa-stained thick blood smear. A sample was considered PCR positive when *P. falciparum* parasite genotyping using standard WHO genotyping procedures yielded a product. A positive RDT result was referred to as RDT positivity results, while a sample was considered positive for *P. falciparum* by RDT when the positive test strip was confirmed by microscopy or PCR. RDT positivity is used frequently as an indication of malaria in some facilities in Ghana where microscopy is unavailable.

### Microscopic estimation of malaria parasite

Thick and thin blood smears as well as dried filter paper blood spots (DBS) were each made from a drop (~50 μl) of finger-prick blood. The blood smears were processed and then stained with 10 % Giemsa for 15 min. The stained slides were subsequently air-dried and viewed under 100X oil immersion microscope. Two independent microscopists read the slides and parasitaemia was determined as the  % of malaria parasite infected RBCs observed per 200 white blood cells (WBCs).

### Extraction of parasite DNA

Genomic DNA was isolated from the membranes of the previously used PfHRP-2 RDT kits and dried filter paper blood spots (DBS) using either Tris–EDTA (TE) [21] or chelex [22]. Briefly, the RDT cassette was opened and portions between the filter paper through to the nitrocellulose membrane and some of the conjugated pad were cut and placed into a 1.5 ml microcentrifuge tube containing 200 μl TE; a separate scalpel was used for each RDT. Similarly, a 3 mm punch was used to punch two 3 mm^2^ disks from each of the dried blood spot (DBS). Each sample pieces was put into a 1.5 ml microcentrifuge tube containing 200 µl TE. The sample tubes were heated at 97 °C for 15 min on a dry heating block, centrifuged at 10,000*g* for 30 s after which the supernatant transferred into a 500 μl tube for storage at −20 °C. For the chelex extraction, 150 μl of 6 % chelex in PBS was added to the tube with the punched DBS disks. The tubes were then incubated at 95 °C for 30 min with intermittent mixing by vortexing followed by a quick centrifugation step. The samples were centrifuged at 6000*g* for 6 min, after which 120 μl of the supernatant was transferred into a 500 µl tube for storage at −20 °C.

### *Plasmodium falciparum* genotyping

The WHO malaria parasite genotyping protocol [23] was followed with slight modifications. PCR reactions were carried out in 15 μl volumes for both the primary and nested reactions. Briefly, the 200 nM M2-0F and M2-0R primers were used to amplify 4 μl of gDNA using One Taq polymerase (NEB). The nested reaction was carried out using 1 μl of the primary PCR product with 200 nM each of the combination of S1Fw/N5rev for the 3D7 type alleles or S1Fw/M5rev for the FC27 type alleles. For GLURP, the G-F3 and G-F4 primer pair was used for the outer PCR reaction and the G-NF and G-F4 primer pair used for the nested inner reaction. All the PCR fragments and the digested products were viewed under UV after resolving on a 2 % agarose gel containing 0.5 μg/ml ethidium bromide. Samples were classified as positive by PCR genotyping if the MSP2 and or GLURP PCR yielded a product following gel electrophoresis.

### PCR-based detection of *pfhrp*2 and *pfhrp*3 genes

The PCR amplification was adapted from Baker et al. [23] with very minor modifications. Briefly, 2 μl of gDNA was used as a template in a 20 μl PCR reaction mixture that contained 200 mM of each primer and 1X AmpliTaq Gold^®^ Fast PCR Master Mix UP. The DNA was initially denatured at 96 °C for 10 min followed by 41 cycles of denaturation at 95 °C for 50 s, annealing at 55 °C for 50 s (*pfhrp*2 gene) or 51 °C (*pfhrp*3 gene) and extension at 68 °C for 1 min. The final extension was performed at 72 °C for 5 min then to 4 °C. Genomic DNA from Dd2.

(*pfhrp*2−), HB3 (*pfhrp*3−) and 3D7 (wild type) were used as controls for the PCR amplifications. The primers used in the amplification of the exon 2 regions of *pfhrp*2 and *pfhrp*3 were *pfhrp*2-F1, *pfhrp*2-F2 and *pfhrp*2-R1 for *pfhrp*2; *pfhrp*3-F1, *pfhrp*3-F2 and *pfhrp*3-R1 for *pfhrp*3, as previously listed [23].

PCR amplification for all samples that gave a negative result for any primer set was repeated using twice the volume of gDNA as template. All PCR amplifications were either nested or semi nested.

### Resolution of PCR amplicons by agarose gel electrophoresis

PCR products were separated by electrophoresis on a 2.0 % agarose gel stained with ethidium bromide in 1X TAE buffer. 10 µl of PCR amplicons were loaded onto the gel, which was run for 1 h at 100 V then observed under UV light. The resolved fragment sizes were determined by comparison with 0.5 μg/μl Gene Ruler 100 bp DNA ladder (Thermo Scientific) loaded on the same gel.

### Data analysis

Crosstab descriptive analysis was performed using IBM SPSS Statistics (version 22). Microsoft Excel was used to draw the table and graphs.

## Results

### *Plasmodium falciparum* parasite carriage by microscopy

**Fig. 1:**
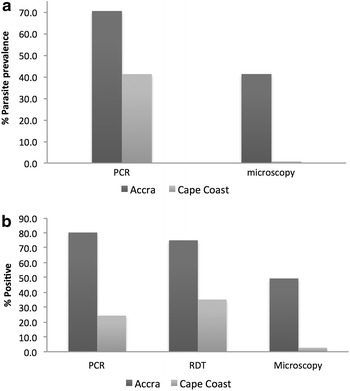
Prevalence of *Plasmodium falciparum* in samples collected in 2015. **a** Giemsa-stained thick blood smears were read and parasite prevalence estimated by microscopy was compared with data obtained from the PCR genotyping of genomic DNA extracted from a DBS. Each Giemsa-stained blood smear analyzed had a corresponding DBS sample. **b** The frequencies of positivity (obtaining a positive test results) obtained by PCR, PfHRP-2 RDT and microscopy in the samples collected in April 2015 from Accra and Cape Coast

Parasite carriage observed by microscopy in Obom (Accra) and Abura (Cape Coast) for February through May of 2015 was 41.2 % (120/291) and 0.7 % (2/267) respectively (Fig. 1a).

### Parasite genotyping by PCR

A parasite carriage rate of 70.4 % (205/291) and 41.2 % (110/267) was identified by PCR genotyping in Accra and Cape Coast respectively during the period of February through May 2015 (Fig. 1a).

In Accra, 46/205 (22.4 %) of the PCR positive samples contained *P. falciparum* parasites that lacked exon 2 of *pfhrp*2. Twenty percent (35/179) of the samples did not yield a product after PCR amplification and were classified as *pfhrp*3− (Table 1). Twenty-six of the PCR-positive samples collected in Accra were omitted from the *pfhrp*3 PCR analysis.

**Table 1:** Prevalence of *pfhrp*2− and *pfhrp*3− parasites in samples collected from February to May 2015

	*pfhrp*2−/*pfhrp*3−	*pfhrp*2−/*pfhrp*3+	*pfhrp*2+/*pfhrp*3−	*pfhrp*2+/*pfhrp*3+
Accra (179)	3.9 % (7)	14 % (25)	16.2 % (29)	65.9 % (118)
Cape Coast (109)	27.5 % (30)	12.8 % (14)	17 % (19)	42 % (46)

*pfhrp*2− no product obtained after *pfhrp*2 exon 2 PCR, *pfhrp*3− no product obtained after *pfhrp*3 exon 2 PCR, *pfhrp*2+ a product was obtained after *pfhrp*2 exon 2 PCR, *pfhrp*3+ a product was obtained after *pfhrp*3 exon 2 PCR. Frequency of occurrence is stated in parenthesis alongside prevalence expressed as a percent of the total population of 179 in Accra and 109 in Cape Coast

In Cape Coast, 40.4 % (44/110) of the PCR positive samples contained *P. falciparum* parasites that lacked exon 2 of *pfhrp2*. Forty-five percent (49/109) of the samples lacked exon 2 of *pfhrp*3. One sample was omitted in the *pfhrp*3 PCR analysis. Overall, 27.5 % of the samples contained parasites that lacked exon 2 of both *pfhrp*2 and *pfhrp*3 (Table 1).

### RDT positivity rate

Two hundred and twenty six (226) spent PfHRP-2 RDT kits were obtained from school children in Accra and Cape Coast in April, 2015. Four of the test kits collected in Accra gave invalid results. An RDT positivity rate of 75 % (114/152) was observed in Accra and 35.1 % (26/74) in Cape Coast (Fig. 1b).

### Malaria estimation by PfHRP-2 RDT

Microscopic examination of Giemsa-stained thick smears made from the same sample spotted onto the PfHRP-2 RDT kit identified *P. falciparum* in 54/152 (35.5 %) and 1/74 (1.4 %) of the samples from Accra and Cape Coast, respectively (Table 2). However PCR analysis of these RDT positive samples confirmed parasites in 94/114 (82.5 %) of the samples from Accra and 7/26 (26.9 %) from Cape Coast (Table 2). False negative RDT results were obtained in 18/38 (47.4 %) of the negative branded RDT kits from Accra and 8/46 (17.4 %) of the negative branded RDT samples obtained from Cape Coast.

**Table 2:** Comparison of microscopy, PCR genotyping and PfHRP-2 RDT results from the samples collected in April 2015

	PCR−	PCR+	Microscopy−	Microscopy+
Accra (RDT−)	31.6 % (12/38)	68.4 % (26/38)	47.4 % (18/38)	52.6 % (20/38)
Cape Coast (RDT−)	75 % (36/48)	25 % (12/48)	97.9 % (47/48)	2.1 % (1/48)
Accra (RDT+)	17.5 % (20/114)	82.5 % (94/114)	52.6 % (60/114)	47.4 % (54/114)
Cape Coast (RDT+)	73.1 % (19/26)	26.9 % (7/26)	96.2 % (25/26)	3.8 % (1/26)

*PCR−* *P. falciparum* negative by PCR genotyping, *PCR+* *P. falciparum* positive by PCR genotyping, *microscopy−* *P. falciparum* negative by microscopy, *microscopy+* *P. falciparum* positive by microscopy, *RDT+* sample produced a positive PfHRP-2 RDT test strip, *RDT−* sample produced a negative PfHRP-2 RDT test strip. A total of 114/152 and 38/152 positive and negative branded RDT kits respectively were collected from Accra and 26/74 positive and 48/74 negative branded RDT kits respectively were collected from Cape Coast

### Contributions of *pfhrp*2 and *pfhrp*3 to malaria diagnosis by PfHRP-2 RDT

Genomic DNA from positive and negative branded PfHRP-2 RDT kits that were confirmed to contain *P. falciparum* by PCR genotyping were subjected to *pfhrp*2 and *pfhrp*3 exon 2 PCR amplification.

**Fig. 2:**
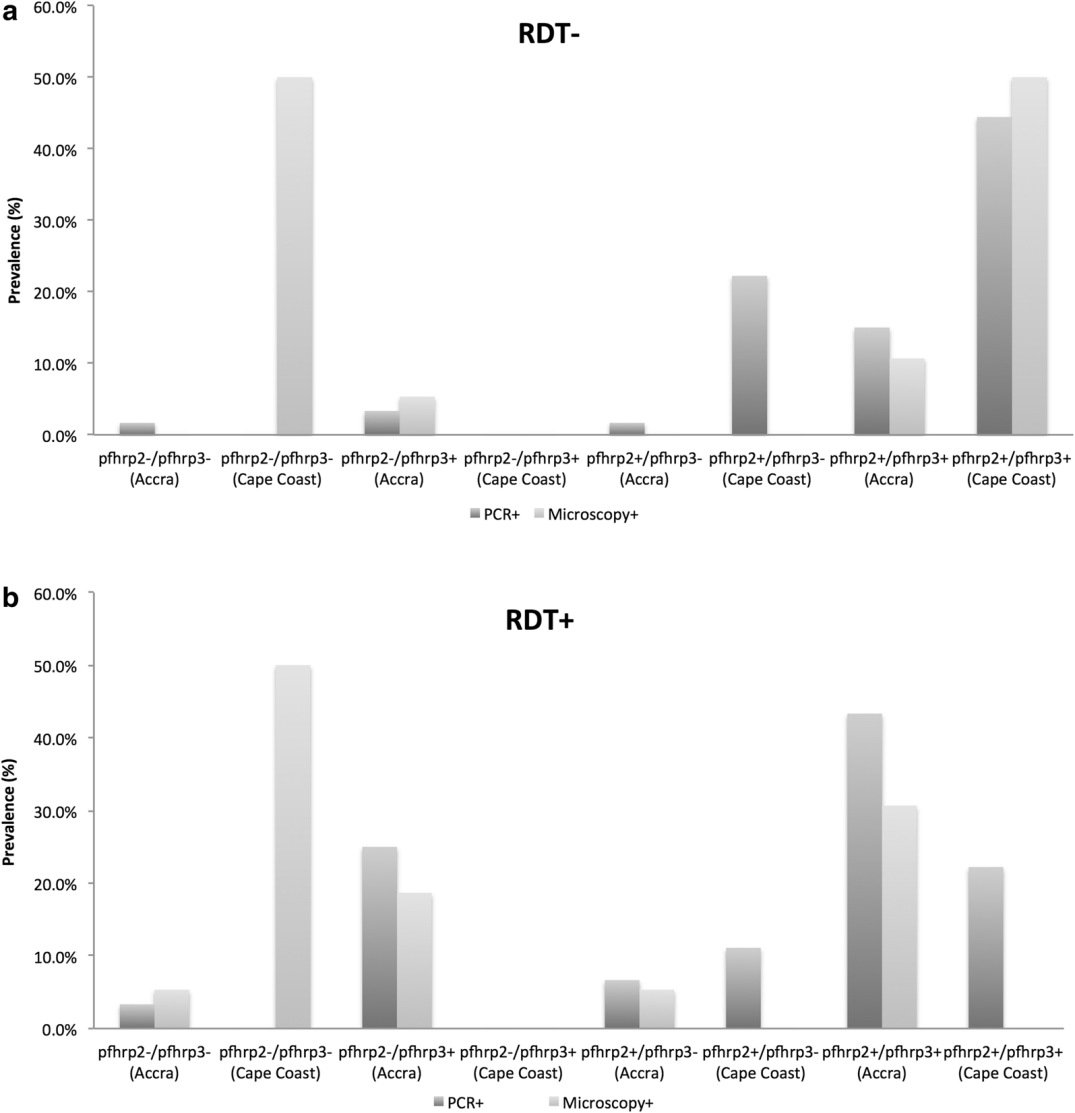
Contributions of *pfhrp*2 and *pfhrp*3 to PfHRP-2 RDT read out. Genomic DNA obtained from either the membrane of the PfHRP-2 RDT kit or the corresponding DBS sample was subjected to *pfhrp*2 and *pfhrp*3 exon 2 PCR amplification. The presence or absence of *pfhrp*2 and or *pfhrp*3 in negative (**a**) and positive (**b**) banded PfHRP-2 RDT kits collected from Accra and Cape Coast was identified. Each RDT kit was confirmed as *P. falciparum* positive by PCR and microscopy

In Accra, 34/94 (36 %) of the PCR-confirmed RDT positive samples lacked *pfhrp*2 (Table 3), 30/34 (88.2 %) of these samples were positive for *pfhrp*3 and 4/34 (17.8 %) of the samples lacked both p*fhrp*2 and *pfhrp*3 (Fig. 2). The prevalence of double *pfhrp*2− and *pfhrp*3− parasites increased from 4.3 % (4/94) to 7.4 % (4/54) in microscopy-confirmed RDT positive samples. The prevalence of *pfhrp*2−/*pfhrp*3+ samples however decreased from 31.9 % (30/94) to 25.9 % (14/54) (Fig. 2). No *pfhrp2*− parasite was identified in any of the RDT positive sample collected in Cape Coast.

**Table 3:** Prevalence of *pfhrp*2− in false negative PfHRP-2 RDT test determined by PCR

	PfHRP-2 RDT−	PfHRP-2 RDT+
pfhrp2 PCR− (Accra)	23 % (6/26)	36 % (34/94)
pfhrp2 PCR+ (Accra)	77 % (20/26)	64 % (60/94)
pfhrp2 PCR− (Cape Coast)	0 % (0/12)	14 % (1/7)
pfhrp2 PCR+ (Cape Coast)	100 % (12/12)	86 % (6/7)

Samples that were confirmed as *P. falciparum* positive by PCR genotyping were grouped according to their PfHRP-2 RDT result, PfHRP-2 RDT positive (PfHRP-2 RDT+) or PfHRP-2 RDT negative (PfHRP-2 RDT−). *pfhrp*2 exon 2 PCR amplification was performed on each sample to estimate effect of the presence of absence of pfhrp2 on malaria diagnosis by PfHRP-2 RDT kits

### Prevalence of *pfhrp*2− and *pfhrp*3− parasites

**Fig. 3:**
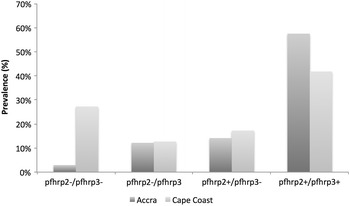
Prevalence of *P. falciparum* parasites lacking exon 2 of *pfhrp*2 and or *pfhrp*3. Samples that were confirmed positive for *P. falciparum* by PCR genotyping were further analyzed for the presence of *pfhrp*2 and *pfhrp*3 by PCR amplification of exon 2. Samples were grouped according to the presence or absence of either or both *pfhrp*2 and *pfhrp*3

In an additional survey of gDNA extracted from DBS, the prevalence of *pfhrp*2− parasites that had intact *pfhrp*3 (*pfhrp*2−/*pfhrp*3+) was to be 14 % (25/205) in Accra and 13 % (14/110) in Cape Coast and the prevalence of parasites with intact *pfhrp*2 that were *pfhrp*3− (*pfhrp*2+/*pfhrp*3−) was 16 % (29/201) in Accra and 17 % (19/109) in Cape Coast (Fig. 3).

## Discussion

The recent recommendation for accurate classification of *pfhrp*2− parasites calls for an initial microscopic evaluation of the parasites, followed by *Plasmodium* species-specific PCR analysis, after which confirmation is carried out by *pfhrp*2 specific gene amplification to determine the absence of the gene or antigen analysis using a second quality PfHRP-2 based RDT or PfHRP-2 based ELISA [24]. Many studies have reported the presence of *pfhrp*2− parasites in a number of South American countries [25–28], one study analysed 68 isolates and did not find any deletions in the *pfhrp*2 gene nor its flanking sequences but rather found 50 % of the isolates to have deletions in the *pfhrp*3 gene and its flanking genes [27]. Such variation between nearby countries raises the need for all malaria endemic countries to engage in nationwide *pfhrp*2 surveillance.

In Ghana, RDTs are used for malaria diagnosis throughout the year, during both peak and off peak seasons. A common practice in a number of health facilities is to rule out malaria in patients that test negative with an RDT kit, without further confirmation. This makes accurate malaria diagnosis using RDT kits very essential for malaria control.

The few previous studies on the PfHRP-2 based RDT kits in Ghana have focused on determining the sensitivity and specificity of Pf-HRP2 RDT kits [17, 29]. This study provides some preliminary evidence for the existence of *pfhrp*2− parasites as well as determines how mutant parasites with deletions in one or both *pfhrp*2 and *pfhrp*3 influence the accuracy of malaria diagnosis by PfHRP-2 RDT in Ghana.

Parasite prevalence estimated by PCR genotyping of 70 % for February to May was almost twice what was estimated by microscopy of corresponding thick blood smears in Accra. In Cape Coast, parasite prevalence estimated by microscopy of 0.7 % was only a small fraction of that estimated by PCR (41.2 %) (Fig. 1a). This suggests that more sensitive diagnostic tools are needed to accurately diagnose malaria in settings with a high prevalence of sub microscopic parasites.

To estimate the true prevalence of *pfhrp*2− parasites, which includes double *pfhrp*2− and *pfhrp*3− (*pfhrp*2−/*pfhrp*3−) parasites; each gDNA sample was analyzed by PCR genotyping prior to *pfhrp*2 and *pfhrp*3 exon 2 PCR.

False positive PfHRP-2 RDT results are not uncommon in malaria endemic settings as the PfHRP2 antigen persists for weeks after parasite clearance; however, in Accra where *P. falciparum* parasite prevalence was high, the PCR estimate of parasite prevalence was comparable to the RDT positive rate (Fig. 1b). Despite the similar diagnostic read out between PCR genotyping and RDT, PCR confirmed the presence of *P. falciparum* in 94/114 of the RDT positive samples, suggesting a false positive rate of 17.5 % (20/114). Microscopic evaluation of the RDT samples increased the false positive rate to 60/114 (52.6 %) in Accra. PCR genotyping identified 26/38 negative branded RDT kits to be positive for *P. falciparum*, out of these 26 samples, 12 were positive by microscopy.

In Cape Coast, the RDT positive rate was higher than parasite estimation by both microscopy and PCR, confirming PfHRP2 antigen persistence. There were only two positive microscopy slides over the entire 4 months (Table 2). The low prevalence and density of *P. falciparum* parasites causes the persistence of PfHRP2 to become more evident.

False negative RDT results are obtained when parasite carriage is confirmed by either microscopy or PCR, however the RDT kit produces a negative test results. This can have severe consequences in malaria endemic settings where negative RDT kit results are not confirmed by any other diagnostic tests such as microscopy. The prevalence of false negative RDT results increased from 18/38 when the samples collected in Accra were confirmed by microscopy to 26/38 when confirmed by PCR (Table 2). This increase was due to PCR confirming more samples as parasite positive than microscopy. Twenty-three percent (6/26) of the false negative samples carried deletions in the *pfhrp*2 gene (Table 3), which suggests other factors including low parasite density contributed more to the negative RDT diagnosis than deletions in *pfhrp*2.

In Cape Coast, PCR genotyping confirmed the presence of 12 false negative RDT tests. All 12 samples were positive for *pfhrp*2 by exon 2 PCR, confirming our observation that factors other than *pfhrp*2 deletion, including the high prevalence of submicroscopic parasites accounted for the false negative RDT results (Table 3).

Persistence of PfHRP-2 antigen from a recent past infection could explain the false positive RDT test, however possible compensation of *pfhrp*3 for the lack of *pfhrp*2 could also contribute to the positive RDT results obtained in samples that lacked *pfhrp*2. Eighty-eight percent of the samples from Accra that were *pfhrp*2− but were positive by PfHRP2 RDT and PCR genotyping were *pfhrp*3+ (Fig. 2). Although the sensitivity for the CareStart™ PfHRP-2 RDT has proven to be very high in relation to microscopy in the recent WHO screen [4, 10] and between 100 and 96 % in Ghana [16, 17]. During the off-peak malaria season, the prevalence of false negative tests was as high as 68.4 % by PCR and 52.6 % by microscopy. The specificity of the CareStart™ PfHRP-2 RDT has previously been found to be between 70 and 73 % in Ghana [16, 17], however the false positive RDT results obtained in Accra were 17.5 % by PCR and 52.6 % by microscopy.

Double *pfhrp*2−/*pfhrp*3− parasites have been found to be as high as 25.7 % in some countries within the Amazon basin [25, 28]. The prevalence of parasites with *pfhrp*2−/*pfhrp*3− was 28 % in samples obtained from Cape Coast over February through May (Fig. 3), however the subset of these samples that were analysed in April did not contain any double *pfhrp*2−/*pfhrp*3− parasite. The prevalence of double *pfhrp*2−/*pfhrp*3− parasites obtained in Accra over the months of February to May was 4.3 %, which was similar to 3 % that obtained in the samples collected in April.

## Limitations

This study was carried out in the off-peak malaria season, where *P. falciparum* prevalence and density is relatively low in most parts of Ghana. Although this is a major limitation, PfHRP-2 RDT kits are used for malaria diagnosis over this period, making this study highly important.

## Conclusion

*Plasmodium falciparum* parasites that lack *pfhrp*2 alone or in addition to *pfhrp*3 have been identified in two regions of Ghana based on the assumption that deletions in exon 2 of these genes represents deletions of the entire gene. Malaria RDT testing is highly suitable for diagnosis in communities where parasite densities are high but become less accurate when parasite densities are low and also where *pfhrp*2 deletant parasites are prevalent. More accurate diagnosis of malaria would be obtained in countries such as Ghana where *pfhrp*2 deletant parasites exits when a more sensitive RDT kit such as the pLDH/PfHRP-2 combo RDT kit that is able to detect *pfhrp*2 deletant parasites is used. More studies are needed to evaluate RDT use in the peak malaria season as well as identify the possible influence *pfhrp*2 and *pfhrp*3 sequence diversity has on the diagnostic read out of PfHRP-2 based RDT kits across Ghana.

## Abbreviations

RDT: rapid diagnostic test
PfHRP-2: *Plasmodium falciparum* histidine rich protein-2
*pfhrp*2: *Plasmodium falciparum* histidine rich protein-2 *gene*
*pfhrp*3: *Plasmodium falciparum* histidine rich protein-3 gene
PCR: polymerase chain reaction
ELISA: enzyme linked immunosorbent assay
*pfhrp*2-: parasite with *pfhrp*2 deleted
*pfhrp*3-: *P. falciparum* with *pfhrp*3 deleted
